# Role of LFA-1 and ICAM-1 in Cancer

**DOI:** 10.3390/cancers9110153

**Published:** 2017-11-03

**Authors:** Manuel Reina, Enric Espel

**Affiliations:** 1 Department of Cell Biology, Physiology and Immunology, Faculty of Biology, University of Barcelona, Diagonal 643, 08028 Barcelona, Spain; 2 Celltec UB, Faculty of Biology, University of Barcelona, Diagonal 643, 08028 Barcelona, Spain

**Keywords:** cancer metastasis, chronic lymphocytic leukemia, exosomes, tumor microenvironment

## Abstract

The lymphocyte function-associated antigen-1 (LFA-1) (also known as CD11a/CD18 and α_L_β_2_), is just one of many integrins in the human body, but its significance is derived from its exclusive presence in leukocytes. In this review, we summarize the studies relating LFA-1 and its major ligand ICAM-1 (or CD54) with cancer, through the function of lymphocytes and myeloid cells on tumor cells. We consider how LFA-1 mediates the interaction of leukocytes with tumors and the role of ICAM-1 in tumor dynamics, which can be independent of its interaction with LFA-1. We also offer a more detailed examination of the role of LFA-1 within B-cell chronic lymphocytic leukemia. Finally, we discuss the role that exosomes harboring LFA-1 play in tumor growth and metastasis.

## 1. Introduction

LFA-1 is widely present in hematopoietic cells, where it mediates intercellular interactions within the immune system and between leukocytes and non-blood cells. Several intercellular adhesion molecules (ICAMs), including ICAM-1 and junctional adhesion molecule 1 are common ligands for LFA-1 [1,2,3]. The strength of LFA-1 adhesion varies according to adjustments in the affinity (conformation), the level of LFA-1 clustering and the force applied by the ligand [4,5,6]. In the most accepted model of activation of LFA-1, the heterodimer can adopt different conformations, from a folded low-affinity inactive conformation to an open active one [7]. As with the other integrins, LFA-1 does not function as a mere adhesive contact between cells, but it also mediates signals that modulate their growth, differentiation and survival. The characteristics of the external ligand binding to LFA-1 initiate a complex signaling cascade within the cell (named “outside-in” signaling), that gives rise to various cellular changes, including promoting the differentiation of naïve T cells to distinct T helper cell subsets [8,9,10,11]. However, this outside-in signaling will not be further discussed in this concise review and readers are referred to other reviews for a detailed description of it [12,13,14].

## 2. Inside-Out Signaling by LFA-1

Here, we present a very schematic view of the flow of signaling from inside the cell to LFA-1 on the cell membrane, known as inside-out signaling, introducing some of the proteins that participate in the activation of LFA-1 adhesion. When leukocytes are stimulated by any of a variety of external stimuli, including antigens and chemokines, the affinity and clustering of LFA-1 increases in the process mentioned above of inside-out signaling, that opens the conformation of LFA-1 and exposes its ligand-binding site [15,16,17]. For example, when T cells are stimulated through engagement with the T cell receptor (TCR), several signaling pathways are initiated, leading to activation of tyrosine kinases and phospholipase C. The best-known connection between the TCR and LFA-1 starts with the activation of the small guanosine triphosphatase (GTPase) Rap1. The phosphorylation of tyrosines recruits the guanine nucleotide exchange factor C3G onto the plasma membrane, which activates Rap1 by converting it from its GDP-bound form to its active GTP-bound form [18]. Another way Rap1 is activated is dependent on phospholipase C, and is mediated by the guanine nucleotide exchange factor CalDAG-GEFI [19,20]. Once activated, Rap1 has several substrates in the pathway linking the TCR signalosome to LFA-1, including the Rap1-interacting molecule (RIAM). RIAM is a crucial adaptor that connects Rap1 to the adaptor talin, recruiting both proteins onto the plasma membrane, where talin is then capable of binding to and regulating the affinity of LFA-1 [21]. This cross-talk between TCR-associated tyrosine kinases and LFA-1 produces a conformational change in the LFA-1 beta chain, increasing its affinity for LFA-1 ligands. Moreover, RIAM and talin are capable of connecting to the actin cytoskeleton, by binding of F-actin and recruitment of the actin nucleation factor Arp2/3. Thus, the Rap1-RIAM-talin complex links actin cytoskeleton dynamics to LFA-1 activation [21]. The capacity of RIAM to regulate actin dynamics has been suggested to contribute also to the outside-in signaling generated as a consequence of integrin adhesion [22].

### 2.1. LFA-1 Participates in the Cytotoxic Immune Response against Tumors

The T cell cytotoxic response against cancer cells starts when the T cell receptor recognizes a specific tumor antigen on the surface of the cancer cell; this is normally followed by delivery of toxic granules that kill the target cell. In the cytolytic response against virus-infected cells, the adhesion between the cytotoxic lymphocyte and the target cell is not strictly dependent on LFA-1, which is, however, necessary in the cytolytic response to immunogenic tumors [23,24]. LFA-1 is an essential initiator of the immunological synapse that forms between the cytotoxic T or NK cell and the cancer cell, and it mediates both firm adhesion to the target cell and the orientation of the cytotoxic granules towards the target [8,25,26]. There is a positive correlation between the maturation state of NK cells, their cytotoxic potential and the activation level of LFA-1 [27]. The amount of LFA-1 on the cell surface of CD8 cytotoxic T cells is additionally regulated by keeping an endosomal pool of LFA-1 [28].

### 2.2. Tumor Infiltrating Lymphocytes Need LFA-1 to Adhere to Target Cells

Cancer cells can escape from the immune response through changes in the tumor microenvironment that make it immunosuppressive [29]. Indeed, the presence of an immuno-suppressive regulatory T (Treg) cell infiltrate within a tumor can correlate with reduced survival [30], but does not if the nature of the T cell infiltrate is inflammatory [31].

Inflammatory tumor-infiltrating lymphocytes (TIL) can be purified from solid tumors and expanded in vitro before being used as an effective therapeutic tool. However, TIL are not fully functional when first obtained from tumors. The presence of a galectin lattice covering the surface of TIL interferes with the adhesion of T cell LFA-1 to ICAM-1 on the target cell [32]. These galectins impair the recruitment and activation of LFA-1 within the immunological synapse formed between TIL and tumor cells [32]. The extent of the LFA-1–ICAM-1 interaction affects the secretion of cytokines by TIL [32].

### 2.3. Leukocytes in the Tumor Stroma Can Play a Positive or Negative Role in Tumor Growth

Cancer cells develop in a dynamically changing microenvironment that constitutes a safe zone for their survival and proliferation. Through the interaction with stromal cells, cancer cells receive survival signals and produce proteins and metabolites that suppress (or otherwise modify) the activity of the immune system. This dependence of the tumor on its microenvironment could also be the tumor’s Achilles heel, so to speak, since tumor-supporting stromal cells could be targeted therapeutically. Myeloid cells can either contribute to tumor development or restraint tumor growth, depending on the tumor context.

What follows is a summary of studies in which LFA-1 has been related to myeloid cell function in the tumor microenvironment. Likewise, we mention the importance of LFA-1 in the function of the Treg that infiltrate tumors.

#### 2.3.1. Neutrophils

In mouse models of breast cancer, neutrophils are the main drivers of metastasis [33]. By producing leukotrienes, neutrophils contribute to the expansion of cancer cells with high tumorigenic potential and thus to metastasis [33]. Hence, targeting the production of leukotrienes in neutrophils has a therapeutic effect on metastasis. In a similar mouse model of estrogen receptor-positive breast cancer, neutrophils were recruited into the tumor via increased expression of LFA-1, provoked by estradiol and TGFβ1 [34]. Neutrophil adhesion to endothelial cells by means of LFA-1 is associated with their prolonged survival [35]. Importantly, neutrophil-cancer cell interactions mediated by LFA-1 facilitated breast cancer cell dissemination in a model of metastasis [34]. In ovarian cancer patients, the adhesive properties of blood neutrophils are increased, due to increased production of the CD11b/CD18 integrin, suggesting that the neutrophil-cancer cell contacts modify the properties of the neutrophils and facilitate tumor dissemination [36].

In contrast, in other types of cancer, neutrophils play a protective role that can act against tumor development [37]. When this is the case, it would be desirable to potentiate neutrophil infiltration into the tumor thereby increasing its destruction. Due to the contrasting roles of neutrophils in cancer, it is necessary to categorize tumors so as to determine the desirability of improving the neutrophil response against the tumor or, in contrast, blocking the help the neutrophil provides the tumor.

#### 2.3.2. Macrophages

Macrophages are an abundant component of many solid tumors and can play varied functions, depending on the phenotype of the macrophage and the tumor context. These tumor-associated macrophages contribute to the epithelial-mesenchymal transition (EMT) of tumor cells and tumor dissemination [38,39,40,41]. The metastasis-promoting effect of macrophages is exerted through secretion of cytokines such as TGFβ [39]. In an in vitro model of EMT, it was shown that M2-type macrophages in direct contact with carcinoma cells facilitated the dispersion of the latter via ICAM-1 and integrin CD18 interaction [42]. Similarly, in a mouse model of ovarian cancer, the initial steps of spheroid formation and transcoelomic metastasis were facilitated by the attachment of cancer cells to macrophages via CD11b/CD18–ICAM-1 adhesion [41]. When binding to ICAM-1 was neutralized with antibodies, spheroid formation and ovarian cancer progression were impaired [41].

#### 2.3.3. Eosinophils

Eosinophils are potential weapons in the anti-tumor arsenal. In a mouse model of melanoma which depends on the anti-tumor activity of cytotoxic CD8 T cells for the survival of the animal, eosinophils played an essential role in the recruitment of cytotoxic T cells into the tumor [43]. In addition, in this melanoma model, eosinophils normalized the tumor vasculature and reprogrammed tumor-associated macrophages into the inflammatory M1 type [43]. Similarly to eosinophils, when basophilia was induced in these mice by treatment with interleukin-13, enhanced T-cell infiltration and tumor rejection resulted, indicating a similar anti-tumor role of basophils [44]. Other studies attribute an anti-tumor role to eosinophils in colon cancer [45]. The cytotoxic activity against tumor cells that eosinophils can display in vitro depends on the interaction between LFA-1 and ICAM-1, which is upregulated by interleukin-18 [46].

#### 2.3.4. Treg Cells

A common cell type that infiltrates solid tumors is the Treg cell, which exerts strong immunosuppressive function. There is a correlation between tumor aggressiveness and the frequency of intratumoral Treg. The Treg cells can be recruited into tumors or differentiate in situ due to the effect of the tumor microenvironment on local T cells [47]. Importantly, Treg cells that infiltrate tumors have a different phenotype than those obtained from normal tissues, and show stronger immunosuppressive activity [48,49,50]. Hence, the phenotypic differences present in intratumoral Treg cells can be used as tools to specifically target these cells and increase the anti-tumoral immunity [51].

LFA-1 is necessary for a proper development and function of Treg cells and when it is absent the propensity for autoimmunity increases [52,53]. Curiously, Treg cells establish stronger adhesions with the dendritic cell than non-Treg cells [54]. These unusual LFA-1-dependent adhesions keep dendritic cells anchored to Treg cells. It remains to be seen whether LFA-1 adhesiveness is also crucial for the function of intratumoral Treg cells. If this were the case, it could be convenient to target LFA-1 in intratumoral Treg cells in order to deactivate them.

### 2.4. LFA-1 in Chronic Lymphocytic Leukemia

Because there are studies that report alterations of the LFA-1 signaling pathway in chronic lymphocytic leukemia (CLL), those studies are being considered here. CLL, the most common leukemia in the Western world, is characterized by the accumulation of clonal mature B cells in blood and lymphoid tissues [55]. Circulating normal B cells continuously home in on secondary lymphoid organs in search of antigens and from where they acquire survival signals. Similarly, B-CLL cells take advantage of this survival path by increasing the expression of the homing chemokine receptors CXCR4 and CCR7, and decreased expression of the egress receptor S1P1, to home in on lymphoid organs: an environment that favors clonal expansion [56]. The concentration of CLL cells and other stromal cells, macrophages and T cells defines a pseudo-follicle, which supports CLL cell proliferation [57]. The importance of migration and adhesion in determining pathogenesis in this leukemia type is exemplified by treatment with ibrutinib, a Bruton’s tyrosine kinase inhibitor, that mobilizes leukemic cells out of the supportive lymphoid organs, thereby resulting in CLL regression [56,58,59]. The inhibition of Bruton’s tyrosine kinase impedes the chemokine-derived and B-cell receptor-derived signaling governing adhesion and migration of CLL cells.

In addition to chemokines, migration of CLL cells requires the participation of the two lymphocyte integrins: VLA-4 (CD49d/CD29) and LFA-1, which show varied levels of expression in CLL cells [60]. VLA-4 contributes to the homing in of CLL cells on bone marrow [61]. However, the regulation of LFA-1 adhesion is impaired in CLL cells, due to defective signaling by Rap1 GTPase, a major signaling element of the inside-out signaling cascade, which impedes proper clustering of LFA-1 [62]. Moreover, signaling by Rac1 and CDC42 GTPases which activates LFA-1 shows extensive degrees of alteration in CLL patients, so that some authors suggest that progression to CLL requires these GTPases to be bypassed [63]. Defective LFA-1-dependent endothelial transmigration could play a role in the survival of CLL cells [64]. Despite the necessity of B-CLL cells to home in on lymph nodes and bone marrow, it is not uncommon to observe a lower CD18 expression in B-CLL than in normal B cells in healthy individuals [60]. Recently, a CD18 variant present in CLL patients has been associated with increased susceptibility to the disease [65]. The variant shows a glutamate-to-lysine E630K change, which probably impairs CD18 function. The expression of the CD18 variant in patients’ B cells is even lower than that of the wild-type form [65]. In contrast, a CLL subgroup of patients harboring trisomy 12 (approximately 16% of CLL patients) show an important increase in LFA-1 and other integrins, which is associated with high cell proliferation and lymph node infiltration [66,67]. These data do not clarify the role of LFA-1 in the progression of CLL and suggest a complex interplay with other adhesion molecules and signaling pathways.

Patients with CLL show impaired T cell function which results from the direct contact of normal T cells with B-CLL cells [68] (reviewed in reference [69]). Thus, the cytotoxic activity of CD8 T cells and the differentiation of T helper 1 are deficient in CLL patients. Inefficient T cell function probably contributes to CLL expansion. These T cells display low conjugation to B-CLL cells in vitro and impaired immune synapses, characterized by impaired LFA-1 clustering at the immune synapse [70]. Importantly, pretreatment of B-CLL cells with anti-ICAM-1 monoclonal antibodies improved both the conjugation of B-CLL cells with T cells and F-actin polymerization at the immune synapse [70]. Furthermore, T cells in CLL patients exhibit defective LFA-1-mediated migration, due to dysregulated Rho GTPase signaling [71]. Treatment with lenalidomide, a clinically active drug used in hematologic cancers [72], restored Rho GTPase signaling in T cells, rescued LFA-1 function [71] and improved immune synapse formation between T and B-CLL cells [70]. It is surprising that T cells in CLL patients show a dysfunctional adhesion and signaling through the LFA-1/Rho-family GTPase pathway, resembling that of B-CLL cells, which highlights the importance of the LFA-1 signaling pathway in this leukemia.

### 2.5. Targeting LFA-1 in Cancer

The specific expression of LFA-1 in hematopoietic cells makes it a potential target in leukemias and lymphomas. Tumors of hematopoietic origin normally express LFA-1, and can be targeted with anti-LFA-1 antibodies [73]. Studies in mice show integrin antagonists successfully block varied inflammatory conditions, however, attempts to functionally antagonize integrins in human tumors have generally failed [74,75]. This is the case of efalizumab, a humanized monoclonal antibody directed against CD11a, which blocks the interaction between LFA-1 and ICAM-1 [76]. Initially used in the treatment of psoriasis, it had to be withdrawn in 2009 after a high incidence of progressive multifocal leukoencephalopathy, due to reactivation of a latent infection by the neurotrophic JC polyoma virus [77]. These results underline the need to better understand the diverse functions of LFA-1. Unforeseen effects when targeting LFA-1 are not uncommon; for instance, the use of antibodies against CD11a/CD18 can indirectly affect the function of the integrin VLA-4, selectively inhibiting it [78]. This shows that the signaling pathways of both integrins are intertwined. So the design and applicability of new LFA-1 antagonists is a potential field for therapy development [79,80].

Instead of blocking LFA-1, there is the possibility of tagging it in order to redirect the host immune system against integrin-bearing cells, by means of an integrin-binding peptide linked to Interleukin-2 and the Fc fragment [81]. The efficacy of this approach depends on the recognition by the immune system of cancer cells labeled with the recombinant protein and their subsequent elimination.

Besides blood cell cancers, expression of LFA-1 has sometimes been reported in non-hematopoietic tumor cells, such as in brain metastasis [82,83]. In a mouse model of a brain tumor, the presence of LFA-1 in metastatic cells made an important contribution to tumor growth [83]. LFA-1 has also been found in in vitro cultured melanoma cells, allowing their transmigration through endothelial cells [84]. However, whether LFA-1 is also expressed in melanoma cells in vivo is unknown. These cases are too uncommon for LFA-1 to be considered a direct target in tumors of non-hematopoietic origin. However, as mentioned above, solid tumors do often contain a leukocyte infiltrate which may play a pro-tumorigenic role. For instance, the presence of Treg cells, which are immunosuppressive, in the microenvironment of many solid tumors, and the importance of LFA-1 for the function of these cells [85,86], suggests that targeting LFA-1 would limit the function of Treg cells and improve the action of the immune system against the tumor [43,44]. The knowledge acquired from targeting LFA-1 in the treatment of inflammatory diseases can help here [7,79]. To our knowledge, though, the targeting of the immunosuppressive leukocyte infiltrate in solid tumors has not yet been attempted.

A different approach is to target the LFA-1 signaling pathway instead of membrane LFA-1. This is more complex since signaling pathways are partially shared between integrins and other cellular receptors. Despite this, the success obtained with some kinase inhibitors is encouraging. This is the case of ibrutinib in the treatment of CLL (see above). The inhibition of Bruton’s tyrosine kinase stops proliferation of leukemic cells and provokes apoptosis [59]. The precise cause of death is unknown, but the detachment of CLL cells from the supportive tissue environment could play a role [87].

The use of modifiers of LFA-1 activity affect also the normal activity of the immune system. For instance, agonists of LFA-1 can strongly influence the fate of T cells, by governing their differentiation to particular T helper cell subsets [10,11] or by programming T cells to become refractory to the immunosuppressive action of TGFβ [88]. The latter results, functionally correlate with an increased differentiation of Treg cells in vitro when LFA-1 is blocked [89]. These important biological effects provoked by signaling derived from LFA-1 have to be taken into account when considering the use of therapeutic tools modifying the activity of LFA-1.

### 2.6. The Effect of Leukotoxin

The liganded and unliganded states of integrins can determine the life or death of cells [90]. It is therefore not surprising that pathogens may utilize these membrane receptors to modulate the immune response. This is the case of leukotoxin: a protein produced by *Aggregatibacter actinomycetemcomitans* that induces apoptosis in leukocytes [91]. It binds to activated LFA-1 and induces apoptosis by several mechanisms [92]. Leukotoxin shows a tendency to kill leukemic cells in an LFA-1-dependent manner [93,94]. Whereas normal hematopoietic cells might be partially sensitive, leukotoxin shows preferential activity against active LFA-1 and spares most blood cells. The death of tumor lymphocytes is caused by a Fas-dependent mechanism [94]. Besides the advantage of counting with a potential therapeutic tool, working out the mechanism behind the action of leukotoxin on LFA-1 leading to cell death will provide new knowledge linking adhesion to cell fate.

### 2.7. The Role of ICAM-1 in Tumors

ICAM-1 is expressed in several tumors, and as a major LFA-1 ligand, it may help in the immunosurveillance process [95,96,97,98,99,100,101,102,103]. Along this line, the presence of ICAM-1 in colorectal cancer has been associated with better prognosis [101,102]. Moreover, the transfection of ICAM-1 into colorectal cancer cell lines inhibits tumor growth and metastasis [104]. Similar observations were obtained from colon epithelium cell lines derived from mice presenting transforming mutations in the *adenomatous polyposis coli* gene, which is mutated in patients affected by familial adenomatous polyposis. These colonic cell lines express ICAM-1, which mediates the interaction with intraepithelial T lymphocytes [105].

The production of prostaglandin E2 in the tumor microenvironment limits the expression of ICAM-1 in tumor cells, reducing the cytotoxic effectivity of T cells [106]. Mouse melanoma tumors that relapse after adoptive T cell therapy show decreased content of ICAM-1 mRNA [107].

Other potential mechanisms by which ICAM-1 could retard tumor cell metastasis have been proposed. The inhibitory effect of cannabinoids on lung cancer cell invasion and metastasis has been suggested to occur via up-regulation of ICAM-1, which then increases the tissue inhibitor of matrix metalloproteinases-1 [108]. It has also been suggested ICAM-1 mediates the differentiation properties of gastrin-releasing peptide on colon cancer cells by enhancing cell–matrix attachment [109].

In contrast, in some reports, the expression of ICAM-1 has been positively correlated with a more aggressive tumor phenotype and metastatic potential [100,110]. For instance, the invasiveness of breast cancer cells has been positively correlated with the expression of *ICAM-1* [111]. Also, it has been suggested that an ICAM-1–ICAM-1 homophilic interaction between breast cancer cells and mesenchymal stem cells in bone marrow mediates the metastatic expansion of cancer cells, displacing hematopoietic stem cells from their niche [112].

Importantly, tumor-associated fibroblasts in colorectal cancer tissue sections also show increased ICAM-1 expression in comparison to healthy mucosa [113]. There is no clear explanation for the apparently contrary roles played by ICAM-1 in tumor development, suggesting that the function of ICAM-1 is context dependent: modulated by the simultaneous action of other membrane receptors. This further complicates the possibilities of using ICAM-1 as a therapeutic target.

### 2.8. Exosomes Carrying LFA-1 and ICAM-1

It is increasingly clear that exosomes released by cancer cells play a key role in cancer progression and metastasis [114,115,116]. The homing in of exosomes released by cancer cells on specific body tissues is mediated by integrins [115]. However, the function of LFA-1 in exosome-directed mutagenesis and metastasis is poorly understood. LFA-1 is present in exosomes released by mast cells, dendritic cells and T cells [117,118,119], and mediates exosome uptake during T cell–dendritic cell contact [118,119,120]. Exosomes harboring ICAM-1 can be captured by LFA-1 present in dendritic cells [121]. ICAM-1-presence in exosomes released by dendritic cells is necessary for stimulation of naive T cells [122,123].

The cellular origin of exosomes may determine their inhibitory or activation function. Thus, exosomes derived from dendritic cells target other recipient dendritic cells via LFA-1–ICAM-1, and increase their capacity to stimulate T cell tumoricidal activity [124]. In contrast, exosomes derived from T cells, when introduced in mice, target dendritic cells via LFA-1 and modulate their function, inhibiting CD4 and CD8 T cell anti-tumoral activity [119,120]. Furthermore, exosomes bearing ICAM-1 that are produced by cancer cells can block adhesion of leukocytes to endothelial cells [125]. In general, the exosomes derived from cancer cells carry immunosuppressive factors that inhibit immune cell functions [126]. Tumor-derived exosomes that are present in plasma have the potential to function as biomarkers for cancer progression [114,126]. Interfering with the function of cancer-derived exosomes is a promising approach to reestablish normal immune cell function.

## 3. Conclusions

In this review, we have commented on studies that report a link between either the integrin LFA-1 or its ligand ICAM-1 and cancer. Leukemic cells that express this integrin can be targeted by anti-LFA-1 drugs. However, targeting membrane LFA-1 comes with the associated problem of cell specificity, due to the expression of LFA-1 in normal leukocytes and the potential for immune dysfunction. Despite the importance of LFA-1 in leukocyte biology, few convenient drugs that target LFA-1 or its signaling pathway have reached the clinical stage.

Meanwhile, despite the fact that tumors of non-hematopoietic origin do not express LFA-1, they are often accompanied by an immunosuppressive microenvironment, which provides them with survival and growth signals. In this context, it is the leukocyte infiltrate supporting the tumor that can be targeted through LFA-1. This is an underexplored approach given the difficulty of categorizing tumors according to the type of leukocyte infiltrate involved.

We have discussed in more detail CLL: a leukemia for which the available information suggests that LFA-1 could be of therapeutic value. In CLL cells, the signaling regulating LFA-1 activity seems to be impaired, limiting CLL cell migration and increasing cell survival. Curiously, LFA-1 activity is also impaired in the T cells of CLL patients, which are dysfunctional. A useful therapy for this leukemia has been derived from an inhibitor of Bruton’s tyrosine kinase: ibrutinib, which blocks essential signaling in CLL cell activation, including the inside-out signaling linking B-cell membrane receptors and chemokine receptors to activation of LFA-1.

As opposed to LFA-1, ICAM-1 is present in several non-hematopoietic tumors where it can play different roles that also seem to be context dependent. This is an added difficulty in the targeting of ICAM-1 for therapeutic use.

Finally, we have introduced the non-trivial role of exosomes harboring LFA-1 or ICAM-1 in cancer. Few data are yet available concerning the importance of LFA-1–ICAM-1 interaction for the function of exosomes in tumor biology. Understanding how cancer cells use exosomes to modulate the immune system will help to prevent their action and promote the anti-tumoral potential of the immune system.
